# Postoperative bleeding complications in breast conserving surgery and the role of antithrombotic medications: retrospective analysis of 4712 operations

**DOI:** 10.1186/s12957-024-03511-5

**Published:** 2024-09-04

**Authors:** Anselm Tamminen, Riitta I. Aaltonen, Marko T. Ristola

**Affiliations:** 1 Department of Plastic and General Surgery, Turku University Hospital, Turku, Finland; 2 University of Turku, Turku, Finland

**Keywords:** Breast-conserving surgery, Antithrombotic medication, Haematoma, Bleeding complications

## Abstract

**Background:**

This study aimed to evaluate the risk and timing of postoperative bleeding complications following breast-conserving surgery (BCS), with or without axillary surgery, especially in relation to perioperative management of antithrombotic medications.

**Methods:**

Data from all patients who underwent BCS for breast cancer between 2010 and 2022 at a single university hospital were collected. Medical records were reviewed for reoperations, unplanned admissions, and patient characteristics.

**Results:**

In total, 4712 breast-conserving surgeries and 3631 axillary surgeries were performed on 3838 patients. The risk of any bleeding complication was 1.1% (40/3571) in breast-conserving surgery, 0.3% (9/2847) in sentinel lymph node biopsy, and 0.5% (4/779) in axillary lymph node dissection. Upon arrival for treatment, 645 (17%) patients were taking antithrombotic medications. The risk of bleeding complications was not elevated in patients whose medication was discontinued at least a day before the surgery (OR 0.84, *p* = 0.76); but it was almost four-fold (OR 3.61, *p* = 0.026) in patients whose antithrombotic medication was continued. However, the absolute risk for bleeding complication was low in these patients as well (2.0%, 15/751). The majority of bleeding complications (85%, 47/55) occurred within 24 h after the surgery.

**Conclusion:**

The risk for bleeding complications was elevated, but still low, after BCS with or without axillary surgery, when antithrombotic medications were continued through the surgical period. Discontinuing antithrombotic medications is not obligatory in these patients.

## Background

Breast-conserving surgery (BCS) is the recommended surgical treatment for breast cancer in most patients. It offers benefits compared to mastectomy, including better quality of life and improved body image post-surgery, without compromising the oncological safety of the treatment [1, 2]. BCS also poses a smaller risk of surgical complications compared to mastectomy [3]. According to previous research, the risk of bleeding complications in breast cancer surgery varies widely but is usually reported to be between 1 and 10%, with mastectomy often having a higher incidence than BCS [4–6]. Consequently, BCS is increasingly utilized also in elderly patients who have comorbidities, who may have an increased risk of postoperative complications [7].

A significant proportion of elderly patients take antithrombotic medications [8, 9]. Traditionally, these medications have been interrupted before the surgery to minimize bleeding during the procedure and to avoid bleeding complications afterwards [10]. Unfortunately, discontinuing the medication predisposes patients to thromboembolic complications. Venous thromboembolism is the second most common cause of death in cancer patients, following the cancer itself, and the risk for thromboembolic complications is especially high in patients with indication for antithrombotic medications [11–13]. The risk of stroke is also increased after surgery, particularly in patients with atrial fibrillation and discontinued anticoagulant therapy. In such patients, the risk of stroke has been estimated to be 0.6%, and up to 6% of all patients who have a stroke have undergone a preceding surgical procedure [14, 15] The risk of any major thromboembolic complications has been evaluated to be 0.4–1.7% [16, 17].

Therefore, the perioperative continuation of antithrombotic medications may prevent serious complications [18, 19]. Unfortunately, because continuation of these medications is expected to complicate the procedure and increase the risk of postoperative bleeding complications, this practice is seldom utilized. It is advised that perioperative antithrombotic management should be based on evaluating the patient’s individual risk for thromboembolism and surgery-related bleeding [20]. This is only possible when surgery-specific information on the risk of complications is available, which warrants detailed research on the topic.

In our hospital, we have increasingly operated on patients undergoing BCS who are on antithrombotic medications without interrupting their treatment. The clinical experience indicates that the risk of bleeding complications is low, but until now, there has been no systematic evaluation of the subject. The present study was conducted to determine how perioperative continuation of antithrombotic medications has affected the risk of postoperative bleeding complications at our tertiary university hospital.

## Methods

The information for all patients who have undergone BCS for breast cancer or ductal carcinoma in situ (DCIS) at a single tertiary hospital (Turku University Hospital) between 2010 and 2022 was collected from the Clinical Informatics Register of Auria. Patients undergoing simultaneous contralateral mastectomy were excluded from the dataset. The data were supplemented from the electronic patient records. The information on medications was recorded before the surgery, and the data was acquired from Auria registers.

The patient characteristics, including indications for antithrombotic medications, details of the surgical procedure, and perioperative management of anticoagulant and antiplatelet medications, were investigated in detail. A patient was considered to have undergone surgery with concurrent antithrombotic (anticoagulant or antiplatelet) medication if they had taken the normal dose of the medication on the day before the surgery.

The medical records were reviewed to detect each bleeding complication after surgery. Reoperations were identified from the hospital’s database using operational codes. All operations initiated by H (for breast surgery), P (for axillary surgery), Q (for skin or subcutaneous tissue surgery) and Z (for reoperation) were evaluated to detect any reoperation associated with the preceding breast surgery. The patient records of all patients diagnosed with T81 (ICD-10 code for surgical complications) were studied for the entire study period. The laboratory data was searched for test results indicating bleeding complications, such as low haemoglobin, haematocrit and blood transfusions. As Turku University Hospital is the only hospital in the Hospital District to treat any major surgical complications, it can be assumed that the number of bleeding complications not detected by this method is very low.

The bleeding complications were classified as.

i) complications requiring reoperation,
ii) any major bleeding complications utilising standardized criteria [21], which in the context of this study was practically defined as surgery-related bleeding involving a decrease of at least 20 g/l in hemoglobin levels, in addition to patients requiring reoperation, and.
iii) any bleeding complication requiring unplanned readmission or hospitalization.

The research protocol of the study was approved by the Hospital District of Southwest Finland (T537/2022).

### Statistical analysis

The data were analysed using JMP 17 Pro (SAS Institute, Cary, North Carolina, USA) analysis software. For patients’ age and body-mass index (BMI), median and interquartile ranges were defined. Frequency tables for bleeding complications were generated. The 95% confidence intervals were defined assuming binomial distribution. Logistic regression analysis was used to identify predictors of postoperative bleeding complications. A chi-square test was performed to categorical variables and a two-sample t-test for normally distributed and Wilcoxon test for non-normally distributed continuous variables. The variables having a relationship *p* < 0.15 were qualified to multivariable logistic regression analysis. In logistic regression analysis, the factors having the highest p-value was eliminated one by one until only factors with statistical significance (*p* < 0.05) were remaining. As a result, the odds ratio (OR) for any bleeding complication was calculated for patients who continued or discontinued antithrombotic medications, using patients without antithrombotic medications as the comparison group.

### Surgical procedure

BCS was performed using monopolar diathermy as the customary instrument. The tumour was removed aiming for 1 cm clinical margins. Sentinel lymph node biopsy (SLNB) was performed using the double technique (preoperative Tc^99m^ isotope injection with lymphoscintigraphy, and intraoperative blue dye injection). Frozen section analysis was performed for all sentinel lymph nodes until 2018, and only for selected cases thereafter, following the updated treatment guidelines. At the beginning of the study period, the pectoral fascia was most often removed during surgery. The practice was gradually abandoned over the course of the study, and in later years, the fascia was systematically removed only if the tumour was located near the pectoral muscle.

When axillary metastasis was detected preoperatively, or metastasis was found in frozen section study, axillary lymph node dissection (ALND) was performed. Until year 2018, frozen section study was performed in each surgery (if no axillary metastasis was detected preoperatively), and after that only in selected patients in accordance with the updated guidelines and results of the Z0011 study. In ALND, surgical energy instruments were usually utilized, most often SonoSurg^®^ (Olympus Corporation, Tokyo, Japan), ThunderBeat^®^ (Olympus Corporation, Tokyo, Japan), LigaSure^®^ (Medtronic, MN, USA), and Harmonic Focus^®^ (Ethicon Inc., Cincinnati, OH, USA). After ALND, a single drain was applied for seven days or until the amount of exudate was less than 80 ml per day.

For wound closure, the breast and subdermal tissue were approximated with absorbable sutures, and the skin was closed with an intracutaneous continuous absorbable suture. Compressive garments were used based on the surgeon’s preference rather than routinely, and their use was not documented.

Patients had a postoperative follow-up appointment approximately three weeks after surgery, when the healing of the wounds was evaluated.

## Results

In total, 3,838 patients were included in the study, with 645 patients taking antithrombotic medications (Table 1).

The antithrombotic medication was discontinued in 299 patients. The majority of these patients (69%, 206 of 299) were taking aspirin for primary prevention, in which case the medication was interrupted for a week before the operation. During the first operation, 295 (7.7%) patients underwent bilateral surgery either for bilateral cancer, diagnostic reasons or to enhance symmetry. After the primary operation, 580 patients (15.1%) underwent repeat BCS due to insufficient surgical margins. Including these reoperations, 4712 breast and 3631 axillary surgeries were performed (Table 2).

One patient underwent bilateral SLNB with no contralateral breast surgery. In six patients, the perioperative management of antithrombotic medications was not explicitly recorded, and these patients were excluded from the analysis. The customary time for discontinuing the antithrombotic medications was seven days for aspirin and other antiplatelet medications, three days for warfarin, and 1–3 days for direct oral anticoagulants (DOAC), which were introduced during the study period.

There were 55 bleeding complications requiring surgical treatment and 15 that were managed without reoperation, totalling 70 bleeding complications. None of the complications not requiring surgical intervention fulfilled the criteria of major bleeding complication, and thus the number of reoperations is the same than the number of major bleeding complications (Table 3). When presenting the results utilizing the conventional Clavien-Dindo Classification system, patients requiring reoperation are classified as class III, while those not requiring reoperation are classified as class I [22].

**Table 1:** Patient characteristics and information of antithrombotic medications

3838 patients	
Age (years), median (IQR)	64.1 (56.6–70.5)
BMI (kg/m^2^), median (IQR)	27.0 (23.9–31.2)
ASA Classification	
ASA Class 1	633 (16.5%)
ASA Class 2	2366 (58.3%)
ASA Class 3	906 (23.6%)
ASA Class 4	48 (1.3%)
Information missing	15 (0.4%)
Bilateral operations	295 (7.7%)
Antithrombotic medication	645 (16.8%)
Discontinued before the surgery	299(7.8%)
Continued over the surgery	340 (8.8%)
- Aspirin	123 (36%)*
- Other antiplatelets	28 (8.2%)*
- Warfarin	76 (22%)*
- Direct oral anticoagulants	61 (18%)*
- Combination therapy (usually aspirin and dipyridamole)	25 (7.4%) *
- Low-molecular weight heparin	27(7.9%)*
Information missing	6 (0.16%)
Patients who later required re-operation due to insufficient margins	580 (15.1%)

Values are n (%) unless otherwise indicated. (IQR = interquartile range, BMI = body-mass index, ASA = American Society of Anesthesiologists. *proportion of all continued antithrombotic medications)

**Table 2:** Details of performed surgical procedures

Number of breast surgeries	4712
BCS utilizing conventional techniques	3571 (75.7%)
BCS utilizing extensive oncoplastic techniques	412 (8.7%)
Repeat BCS due to insufficient margins in primary operation	580 (12.3%)
Contralateral breast reduction surgery	149 (3.2%)
Indication for breast surgery	
Breast cancer	4026 (85.4%)
DCIS	460 (9.8%)
Diagnostic/benign	41 (0.9%)
Symmetric	185 (3.9%)
Axillary procedure	
SLNB	2851 (60.5%)
ALND	780 (16.5%)
None	1082 (23.0%)
Operation time in minutes (median, IQR)	84 (63–108)

Values are n (%) unless otherwise indicated. (SLNB = sentinel lymph node biopsy, ALND = axillary lymph node dissection, BCS = breast conserving surgery, DCIS = ductal carcinoma in situ, IQR = interquartile range)

In statistical analysis, the variables having *p* < 0.15 were year of the operation, use of antithrombotic medications and the molecular subtype of the cancer. Patients age, ASA Classification, duration of the surgery and performed axillary surgery were not associated with the bleeding complications. BCS’s were performed by 37 surgeons, with individual surgeons having no association to bleeding complications (*p* = 0.96).

In multivariate analysis, patients whose antithrombotic medication was discontinued presented no increased risk for bleeding complications, but patients undergoing the operation with concurrent antithrombotic medication presented a nearly four-fold increase. The relative risk of bleeding appears to be higher in axillary surgery than in breast surgery (Table 4).

**Table 3:** Number of bleeding complications

Number of bleeding complications	All patients	Patients not consuming antithrombotic medications	Patients consuming antithrombotic medications	
**Breast surgery**			Medication discontinued	Medication continued
BCS utilizing conventional techniques				
-requiring reoperation -all complications	0.9% (32/3571) 1.1% (40/3571)	0.9% (28/2956) 1.2% (34/2956)	0% (0/287) 0.3% (1/287)	1.2% (4/324) 1.9% (6/324)
BCS utilizing extensive oncoplastic techniques				
-requiring reoperation - all complications	1.5% (6/411) 1.7% (7/411)	1.4% (5/360) 1.7% (6/360)	6.3% (1/16) 6.3% (1/16)	2.9% (1/35) 2.9% (1/35)
Repeat BCS due to insufficient margins				
-requiring reoperation - all complications	0.7% (4/580) 1.2% (7/580)	0.6% (3/490) 0.8% (4/490)	0% (0/46) 4.3% (2/46)	2.3% (1/44) 2.3% (1/44)
Contralateral breast reduction surgery				
-requiring reoperation - all complications	2.0% (3/148) 2.0% (3/148)	1.6% (2/122) 1.6% (2/122)	0% (0/11) 0% (0/11)	6.7% (1/15) 6.7% (1/15)
**Axillary surgery**				
SLNB				
-requiring reoperation - all complications	0.2% (7/2847) 0.3% (9/2847)	0.2% (4/2395) 0.2% (5/2395)	0.5% (1/204) 0.5% (1/204)	0.8% (2/248) 1.2% (3/248)
ALND				
-requiring reoperation - all complications	0.4% (3/779) 0.5% (4/779)	0.2% (1/642) 0.2%(1/642)	0% (0/72) 0% (0/72)	3.1% (2/65) 4.6% (3/65)

Values are given as percentage (number of complications / total number of patients). Six patients with missing data regarding the perioperative management of antithrombotic medication are excluded from the data. (SLNB = sentinel lymph node biopsy, ALND = axillary lymph node dissection, BCS = breast conserving surgery, DCIS = ductal carcinoma in situ, IQR = interquartile range)

However, when the bleeding risk between BCS and axillary surgery patients was compared using Fisher’s exact test, the difference was not significant (*p* = 0.1344). Due to the low absolute number of complications in axillary surgery patients, a meaningful statistical multivariance analysis in terms of odds ratio could not be performed separately for these patients. Among the patients undergoing surgery with antithrombotic medication, there was no difference in bleeding risk antithrombotic medications were compared to each other (*p* = 0.80).

**Table 4:** Odds ratio for any bleeding complication in relation to antithrombotic medications

	OR	95% confidence interval	*p*-value
Continued antithrombotic medication	3.61	1.17–11.11	*p* = 0.026
Discontinued antithrobotic medication	0.85	0.30–2.38	*p* = 0.76
No medication (reference)	1.00		

(OR = odds ratio)

In total, 47 patients experienced a bleeding complication right after the surgery and underwent prompt reoperation (Table 5).

**Table 5:** Details of the timing when reoperations were performed

Timing of bleeding complications	*n* (%)
The day of the primary operation	27 (49%)
1. postoperative day	17 (31%)
2. postoperative day	3 (5.5%)
Postoperative days 3–7	0
Postoperative days 8–20	5 (9.1%)
Postoperative days 21–49	3 (5.5%)

All the 47 bleeding complications which required reoperation during the first two postoperative days were diagnosed within 24 h of the surgery

Each of these complications was detected within 24 h of the operation, but several patients had to wait to return to the operating room due to more urgent surgical patients taking precedence. None of these patients were discharged before the bleeding complication was detected. No patient was diagnosed with a bleeding complication 3–7 days after the surgery, but eight patients presented with late bleeding complications 8–49 days post-surgery, three of whom were taking antithrombotic medications. Two of the late complications were preceded by a seroma puncture, and three were associated with physical exercise or strenuous movements predisposing the bleeding. In three patients, the hematoma had apparently developed slowly after the surgery and detected only when the patient had the postoperative follow-up visit.

Adjuvant treatment was delayed for two patients: one who developed a surgical site infection after hematoma evacuation and another who experienced the complication on the 49th postoperative day following seroma puncture. In both cases, adjuvant treatment was postponed by 3–4 weeks. Neither of these patients was taking antithrombotic medications. In a separate case, adjuvant treatment was delayed for a week; however, this patient required reoperation due to insufficient resection margins, and it is unclear which factor was the predominant for the delay. This patient was on warfarin, which was continued throughout both surgeries.

Most patients requiring reoperation demanded a single additional day in the surgical ward. Patients whose complications were detected at the end of the weekdays (9 patients) were typically hospitalized over the weekend after the reoperation. Patients who were planned to be hospitalized for the first postoperative night and underwent reoperation on the same day as the primary operation were generally discharged on the first postoperative day as originally planned.

Five of the patients with bleeding complications received blood cell transfusions. All these patients also underwent reoperation.

Patients treated with a conservative approach typically required one additional emergency room admission and a single puncture, except one patient who was punctured four times.

## Discussion

The rationale for interrupting antithrombotic medication is to reduce the number of bleeding complications. In our material, while bleeding complications were statistically almost four times as likely if antithrombotic medication was continued, the overall incidence of bleeding complications was still relatively low. The overall risk for bleeding related reoperations was 0.9%, which is lower than in most research previously published [4–6].

Furthermore, although the complications may have caused significant discomfort, none posed any significant risk to the patients’ general health. The vast majority of bleeding complications were detected during the initial hospital stay, and the affected patients underwent prompt reoperation. In many cases, this did not prolong the hospital stay beyond the originally planned duration. Only in two cases was adjuvant therapy delayed, and neither of these patients was taking antithrombotic medications.

Out of the 55 patients requiring reoperation for a bleeding complication, 8 (15%) were operated on between postoperative days 8 and 49. It can be argued that these late complications could not be avoided by interrupting antithrombotic medications perioperatively.

While a bleeding complication after BCS can be considered a non-life-threatening complication, a thromboembolic event caused by interrupting antithrombotic medications perioperatively, on the other hand, may be fatal [23]. It has been shown that discontinuing antithrombotic medication significantly increases the risk of thromboembolic events, especially in patients with a history of deep vein thrombosis or pulmonary embolism, and continuing the medication decreases that risk [24, 25]. Patients with previous cerebrovascular events have an increased risk of stroke after surgery, especially when the medication is interrupted perioperatively [15, 18, 19, 26]. Therefore, it appears to be a worthwhile goal to continue antithrombotic medications in all patients where it is deemed safe.

Continuing antithrombotic medication routinely simplifies perioperative protocol and reduces the risk of medication errors, which is increasingly beneficial as the population grows older and antithrombotic medication among patients become more common [8, 9, 20]. Additionally, some guidelines may suggest that if antithrombotic medication was supposed to be discontinued for surgery but was inadvertently continued, the surgery should have been postponed. The results of the present study indicate that in such cases, it would be safe and preferable to proceed with the operation as planned. Further research and discussion on the safe continuation of antithrombotic medication perioperatively is warranted, but based on these results it seems that the benefits of continuing the medication, at least in patients with previous thromboembolic events, probably outweighs the risks.

In the present study, we have demonstrated that continuing antithrombotic medications perioperatively in BCS is relatively safe, with no serious or permanent adverse effects on the patient. Whether discontinuing the medication for 1–2 days would be beneficial depends on the risk of major thromboembolic complications after a short interruption. Since antithrombotic medications are used for various indications and have different pharmacological properties, this matter should be further investigated individually for each medication and indication.

This study has several limitations. First, due to the retrospective nature of the study, there may be missing information. Although the patient records were scrutinized for antithrombotic medications and for the perioperative management of these drugs, it is possible that patients have not followed the instructions given before the surgery. Thromboembolic complications were not recorded, which would be desirable to weigh pros and cons of pausing the antithrombotic medications. Further study on this issue is warranted. Furthermore, the limited number of complications leads to uncertainties in the statistical analysis, especially considering axillary surgery. This should be given special attention when interpreting the results.

## Conclusions

Although continuing antithrombotic medications increases the risk of bleeding complications in breast-conserving surgery, with or without axillary surgery, the absolute risk of these complications is low. Additionally, the consequences of bleeding complications are generally tolerable, with no serious adverse effects. Therefore, discontinuing antithrombotic medications does not seem to be necessary in these patients, especially when the risk of thromboembolic events is considered high.

## Data Availability

The data that support the findings of this study are available on request from the corresponding author. The data are not publicly available due to privacy or ethical restrictions.

## Abbreviations

ALN: Axillary lymph node dissection
BCS: Breast-conserving surgery
BMI: Body-mass index
DCIS: Ductal carcinoma in situ
DOAC: Direct oral anticoagulant
SNLB: Sentinel lymph node biopsy
OR: Odds ratio
